# Preoperative serum HER2 extracellular domain levels in primary invasive breast cancer

**DOI:** 10.1186/1471-2407-14-929

**Published:** 2014-12-10

**Authors:** Sae Byul Lee, Jong Won Lee, Jong Han Yu, Beom Seok Ko, Hee Jeong Kim, Byung Ho Son, Gyungyub Gong, Hee Jin Lee, Sung-Bae Kim, Kyung Hae Jung, Jin-Hee Ahn, Woochang Lee, Joohon Sung, Sei-Hyun Ahn

**Affiliations:** Department of Surgery, University of Ulsan College of Medicine, Asan Medical Center, 388-1 Pungnap 2 dong, Songpa-Gu, Seoul, 138-736 Korea; Department of Pathology, University of Ulsan College of Medicine, Asan Medical Center, Seoul, Korea; Department of Oncology, University of Ulsan College of Medicine, Asan Medical Center, Seoul, Korea; Department of Laboratory Medicine, University of Ulsan College of Medicine, Asan Medical Center, Seoul, Korea; Department of Epidemiology, School of Public Health and Institute of Health and Environment, Seoul National University, Seoul, Korea

**Keywords:** HER2 extracellular domain, HER2 breast cancer, Prognostic factor

## Abstract

**Background:**

Despite the preclinical outcomes and biologic significance of the presence of the human epidermal growth factor receptor-2 (HER2) extracellular domain (ECD), there is little evidence supporting the measurement of ECD levels in any clinical setting. The aim of this study was to determine the prevalence of elevated serum HER2 ECD levels, the association between these levels and tissue HER2 overexpression, and the potential clinical prognostic value of HER2 ECD in primary invasive breast cancer.

**Methods:**

Serum HER2 ECD levels were examined preoperatively in 2,862 consecutive stage I–III primary breast cancer patients between 2007 and 2009. Serum HER2 ECD levels were measured by chemiluminescence immunoassay (ADVIA Centaur), and the tissue HER2 status was assessed by immunohistochemistry and fluorescence *in situ* hybridization. The cutoff value for the serum level of HER2 ECD was set at 15.2 ng/ml.

**Results:**

Among the 2,862 patients, 126 (4.4%) had elevated serum HER2 ECD levels, and HER2 was overexpressed in the tumor tissue of 692 patients (24.2%), with a concordance of 78.7%. Multivariate analysis revealed that elevated serum HER2 ECD was a significant independent prognostic factor for worse distant-metastasis-free survival [DMFS; hazard ratio (HR) = 2.50, 95% confidence interval (CI) = 1.5–4.3, *P* = 0.001] and breast-cancer-specific survival (BCSS; HR = 2.0, 95% CI = 1.1–3.8, *P* = 0.036), which were much stronger in patients with tissue HER2-positive tumors (DMFS: HR = 3.8, 95% CI = 2.0–7.0, *P* < 0.001; BCSS: HR = 2.6, 95% CI = 1.2-5.3, *P* = 0.012).

**Conclusions:**

Given the prevalence of HER2 expression, its measurement as an independent prognostic factor can be clinically useful, particularly in patients with tissue HER2-positive tumors.

## Background

Human epidermal growth factor receptor-2 (HER2) overexpression or amplification in tumor tissue is reportedly observed in 20–30% of primary breast cancers. Tissue HER2 status is now routinely used as an important parameter for decision making regarding anti-HER2 therapy in the neoadjuvant [1], adjuvant [2], and metastatic [3] settings. The presence of HER2 in tumor tissues also adds predictive and prognostic information regarding shorter overall and disease-free survivals [4], preferential benefit from doxorubicin [5–7], and possible resistance to tamoxifen [8–10]. However, despite the continuous and varying degrees of tissue HER2 expression, tumors are arbitrarily dichotomized into HER2-positive and HER2-negative groups based on immunohistochemistry (IHC) and fluorescence *in situ* hybridization (FISH) [11]. As a result, the definition of tissue HER2 positivity remains a matter of controversy [12–14]. In addition, there is recent evidence that some tissue HER2-negative patients appear to benefit from trastuzumab [12, 15].

Serum HER2 extracellular domain (ECD) can be detectable by the cleavage of transmembrane tissue HER2 protein which is probably mediated by a matrix metalloproteinase [16–18]. Given the same origin of each HER2 subcomponent in tissue and blood, the technical problems inherent in current tissue HER2 testing methods have urged the exploration of clinical utilities of serum HER2 ECD [19]. The quantification of serum HER2 ECD in primary breast cancer could theoretically enhance the sensitivity of tissue HER2 testing in the minority population in which HER2 expression is significant, but is not sufficiently high to be considered as HER2-positive by the American Society of Clinical Oncology (ASCO) and College of American Pathologists (CAP) guidelines, possibly due to tumor heterogeneity [11, 20–22]. In the metastatic setting, the findings of some studies further suggest that HER2 ECD levels and changes therein reflect the patients’ responses to antiestrogen therapy [23, 24], chemotherapy [25, 26], and trastuzumab [27, 28]. There have been a few positive results in the adjuvant setting, similar to the metastatic setting [29–33], but most studies have addressed the relationship between abnormal HER2 ECD levels and tissue expression positivity with contradictory results. The current consensus is that although preoperative HER2 ECD appears to be correlated with tumor size and nodal involvement, it may not be related to tissue HER2 status, especially in primary breast cancer, and there is insufficient evidence to support incorporating measurement of this parameter into the routine clinical management of women with breast cancer as an independent prognostic factor [14, 34].

The aims of the present study were to establish the prevalence of elevated HER2 ECD levels, and determine whether there is an association between serum HER2 ECD levels and tissue HER2 overexpression in a large number of patients with primary breast cancer, using a single assay approved by the US Food and Drug Administration (FDA). Furthermore, we sought to elucidate differences in any such association between various subgroups of patients, as reported previously [35, 36], and to determine the prognostic usefulness of HER2 ECD and derive evidence for verifying previous equivocal reports [14, 34].

## Methods

Patients who were diagnosed with and had surgery for stage I–III primary breast cancer at Asan Medical Center between January 2007 and December 2009 were enrolled for this study (*n* = 2,862). Patients with distant metastasis at the time of diagnosis, bilateral cancer, and the initial plan of neoadjuvant systemic therapy were excluded. All of the patients’ information and tumor characteristics were retrieved from our prospectively collected database. The size of the tumor, regional lymph node (LN) status, histologic grade, nuclear grade, presence of lymphovascular invasion (LVI), histological subtype, and immunohistochemical status of estrogen receptors, progesterone receptors, and HER2 were determined at our institute by pathological analysis on formalin-fixed paraffin-embedded tissue sections of the primary tumor excised at the time of definitive surgery. Tumor staging followed the tumor-node-metastasis classification of the 7th American Joint Committee on Cancer [37]. This study was reviewed and approved by the Institutional Review Board of Asan Medical Center (20141162).

### Tissue HER2 immunohistochemistry

Formalin-fixed, paraffin-embedded sections (4 μm in thickness) were deparaffinized, dehydrated through a graded alcohol series, and subjected to blocking with hydrogen peroxide and allowed to dry for 10 min at RT, followed by 20 min in an incubator at 65C. IHC was performed in a BenchMark XT autostainer (Ventana Medical Systems, Tucson, AZ) using OptiView DAB Detection Kit (Ventana Medical Systems) for HER2 (cat. 800–4422, clone 4B5, dilution 1:8, Ventana Medical Systems, Tucson, AZ, USA).

The results were graded according to the level of coloring of cell membrane of cancer cells. The cases where less than 10% of the tumors cells stained positively were graded as 0, cases where membrane staining was partial but occurred in greater than 10% of the tumor cells were scored as 1+, cases where entire cell membranes stained modestly were graded as 2+, and cases where entire cell membranes stained strongly but occurred in greater than 30% of the tumor cells were graded 3+. Cases graded 3+ were automatically considered positive, while tumors graded 2+ were further evaluated by fluorescence *in situ* hybridization (FISH) using the Abbott PathVysion *HER2* DNA Probe Kit protocol (Abbott Laboratories, Abbott Park, Des Plaines, USA), with additional monitoring for the progress of proteolytic digestion by propidium iodide staining. Probe mixes were hybridized at 37°C between 14 and18h. After hybridizations, slides were washed in 2 × SSC/0.3% NP-40 at 72°C for 30 min, air dried, and counterstained with DAPI. The results were reported as the ratio between the average copy number of the *HER2* gene and that of the chromosome 17 centromere, analyzing 20 neoplastic nuclei. Specimens with a signal ratio of <1.8 were considered negative for *HER2* gene amplification, whereas those with a signal ratio > 2.2 were considered positive for *HER2* gene amplification. If a signal ratio fell on or between the values of 1.8 and 2.2, we counted the number of signals in an additional 20 nuclei in a second target area. The signal ratio was then calculated from both target areas (40 cells).

### Serum HER2 ECD assay

Serum samples were obtained from breast cancer patients at our cancer center at the time of cancer diagnosis; 5 ml of blood was drawn into serum-separation tubes before surgery and then subjected to chemiluminescence immunoassay. The serum HER2 ECD test is a two-site sandwich immunoassay using two monoclonal antibodies that combine specifically with the HER2 ECD through direct chemiluminescence (ADVIA Centaur System, Siemens Healthcare, Tarrytown, NY, USA). Measurements were performed strictly according to the manufacturer’s instructions and quality control was ensured. The serum HER2 ECD assay for each patient was performed as part of the routine preoperative work-up procedures at the Department of Laboratory Medicine, and the results were stored in our database. We used the manufacturer’s recommended cutoff value of 15.2 ng/ml.

### Statistical analysis

Distant-metastasis-free survival (DMFS) was defined as the time from surgery to the first appearance of distant metastasis. Breast-cancer-specific survival (BCSS) was defined as the time from surgery to the time of breast-cancer-specific death. Correlations between elevated serum HER2 ECD and several variables were evaluated using the chi-square test, and the means of continuous variables such as age and serum HER2 ECD among different groups were compared using ANOVA. Survival curves were generated using the Kaplan-Meier method, and the significance of survival differences among selected variables was verified using the log-rank test. The Cox proportional-hazards model was used to evaluate the independent prognostic effect of serum HER2 ECD on DMFS and BCSS. Unless stated otherwise, the data are presented as mean ± SD, and the cutoff for statistical significance was set at *P* < 0.05. All statistical analyses were performed using SPSS version 12.0 (SPSS, Chicago, IL, USA).

## Results

### Patient characteristics

The age of the entire cohort (*n* = 2,862 patients) was 48.5 ± 9.9 years (range, 22–94 years), and the numbers of patients at stages I, II, and III were 1,245 (43.5%), 1,212 (42.3%), and 405 (14.2%), respectively. LN metastasis was detected in 1,113 patients (38.9%), and positive hormone-receptor status was found in 1,940 patients (67.8%). Tissue HER2 positivity was detected by IHC and FISH in 692 patients (24.2%), among which 264 patients (38.2%) received adjuvant anti-HER2 therapy with trastuzumab. The details of the patient characteristics are summarized in Table 1.

**Table 1:** **Distributions of serum human epidermal growth factor receptor-2 (HER2) extracellular domain (ECD) levels and proportions of patients with elevated serum HER2 ECD levels (i.e., >15.2 ng/ml) according to subgroups stratified according to various clinicopathologic variables (** ***n*** **= 2,862)**

Subgroup classified by variables	*n*(%)	Serum HER2 ECD level (ng/ml)		*P* ^a^	Patients with HER2 ECD >15.2 ng/ml	
		Mean ± SD	Median (range)		*n*	% ^b^
Total patients	2,862 (100%)	10.1 ± 9.4	9.2 (3.3–427.8)		126	4.4%
Age, years				0.149		
≤ 34	172 (6%)	10.1 ± 5.6	8.8 (4.8–56.0)		10	5.8%
35–49	1,510 (53%)	9.8 ± 12.1	8.8 (3.3–427.8)		55	3.6%
≥ 50	1,180 (41%)	10.5 ± 9.4	9.8 (4.4–90.1)		61	5.2%
Stage				<0.001		
I/II	2,457 (86%)	9.6 ± 4.6	9.1 (3.3–184.3)		81	3.3%
III	405 (14%)	12.8 ± 22.1	10.1 (5.5–427.8)		45	11.1%
Tumor size, cm				0.001		
≤ 2	1,676 (59%)	9.6 ± 5.1	9.0 (3.3–184.3)		51	3.0%
> 2	1,186 (41%)	10.8 ± 13.2	9.5 (3.8–427.8)		75	6.3%
Lymph node				<0.001		
Negative	1,749 (61%)	9.6 ± 3.0	9.0 (3.3–48.2)		55	3.1%
Positive	1,113 (39%)	11.0 ± 14.6	9.4 (4.3–427.8)		71	6.4%
Histologic grade						
1/2	1,787 (64%)	9.7 ± 5.5	9.0 (3.8–184.3)	0.002	53	3.0%
3	1,025 (36%)	10.8 ± 13.8	9.5 (4.6–427.8)		71	6.9%
Unknown	50				2	
Nuclear grade				0.006		
1/2	1,775 (63%)	9.6 ± 5.4	9.0 (3.3–184.3)		45	2.5%
3	1,049 (37%)	10.6 ± 13.6	9.4 (4.6–427.8)		65	6.2%
Unknown	38				16	
LVI				0.018		
Negative	2,095 (76%)	9.8 ± 3.7	9.1 (3.3–67.5)		88	4.2%
Positive	660 (24%)	10.3 ± 8.5	9.3 (3.8–184.3)		29	4.4%
Unknown	107				9	
Hormone-receptor status				<0.001		
Negative	916 (32%)	11.5 ± 15.0	9.8 (4.6–427.8)		85	9.3%
Positive^c^	1,940 (68%)	9.5 ± 4.7	9.0 (3.3–184.3)		41	2.1%
Unknown	6				0	
Tissue HER2 status				<0.001		
Negative	2,168 (76%)	9.3 ± 4.3	8.9 (3.8–184.3)		22	1.0%
Positive^d^	692 (24%)	12.8 ± 17.3	10.6 (3.3–427.8)		104	15.0%
Unknown	2				0	
Definitive surgery				<0.001		
Conservation	1,781 (62%)	9.3 ± 2.5	8.9 (3.3–44.8)		38	2.1%
Mastectomy	1,081 (38%)	11.4 ± 14.9	9.7 (4.3–427.8)		88	8.1%
Radiotherapy				0.135		
Yes	2,047 (72%)	9.9 ± 10.2	9.1 (3.3–427.8)		74	3.6%
No	813 (28%)	10.5 ± 7.1	9.6 (4.3–184.3)		52	6.4%
Unknown	2				0	
Chemotherapy				<0.001		
Yes	1,852 (65%)	10.6 ± 11.5	9.4 (4.3–427.8)		106	5.7%
No	1,007 (35%)	9.2 ± 2.6	8.9 (3.3–35.6)		20	2.0%
Unknown	3				0	
Anti-HER2 therapy				<0.001		
Yes	264 (9%)	15.0 ± 27.2	11.0 (4.3–427.8)		53	20.1%
No	2,598 (91%)	9.6 ± 4.4	9.1 (3.3–184.3)		73	2.8%

^a^Calculated by ANOVA.

^b^Percentage calculated by dividing the number of patients with elevated serum HER2 ECD level (>15.2 ng/ml) by the total number of patients in each subgroup.

^c^Estrogen- and/or progesterone-receptor positive.

^d^Graded as 3+ on immunohistochemistry (IHC) or 2+ on fluorescence *in situ* hybridization in cases of IHC 2+.

*LVI* lymphovascular invasion.

### Distribution and prevalence of preoperative serum HER2 ECD levels in primary breast cancer

**Figure 1:**
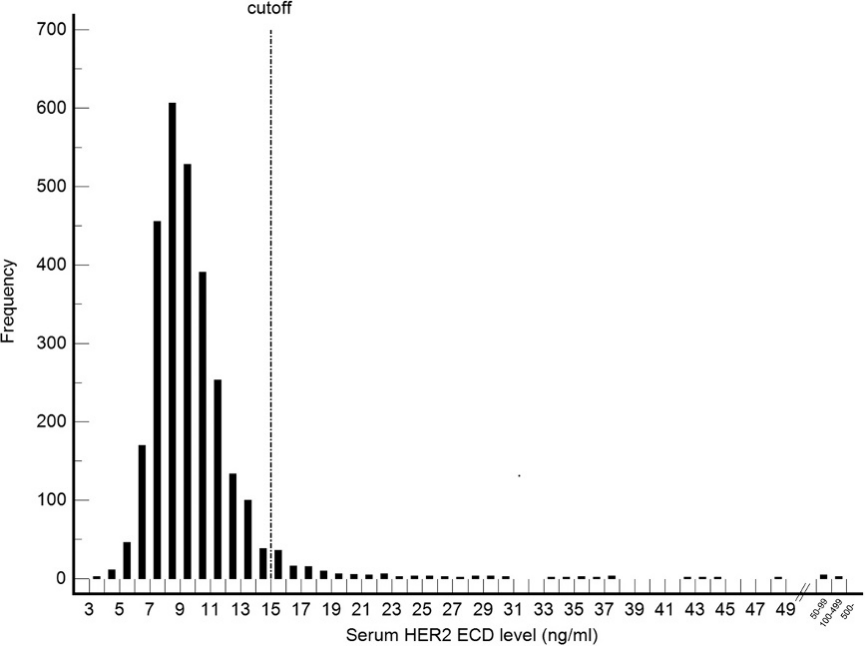
**Histogram showing the frequencies of serum human epidermal growth factor receptor-2 (HER2) extracellular domain (ECD) levels (ng/ml) in 2,862 patients with primary invasive breast cancer.**

The HER2 ECD level before surgery was 10.1 ± 9.4 ng/ml (Table 1); 2,736 patients (95.6%) had an HER2 ECD level of ≤15.2 ng/ml, while in 126 (4.4%) the HER2 ECD level exceeded that cutoff value (Table 1 and Figure 1). The HER2 ECD levels in the normal and elevated HER2 ECD groups were 9.3 ± 1.9 and 27.3 ± 40.4 ng/ml, respectively (Table 2).

**Table 2:** **Baseline characteristics and association between dichotomized serum HER2 ECD level and clinicopathologic variables**

Characteristics	Total group		Subgroup with normal serum HER2 ECD level (≤15.2 ng/ml)		Subgroup with elevated serum HER2 ECD level (>15.2 ng/ml)		*P* ^a^
	*n*	%	*n*	%	*n*	%	
Total patients	2,862	100%	2,736	100%	126	100%	
Serum HER2 ECD, ng/ml							
Mean ± SD	10.1 ± 9.4		9.3 ± 1.9		27.3 ± 40.4		<0.001^b^
Median (range)	9.2 (3.3–427.8)		9.1 (3.3–15.2)		18.5 (15.3–427.8)		
Age at diagnosis, years							0.860^b^
Mean ± SD	48.5 ± 9.9		48.5 ± 9.9		48.4 ± 10.3		
Median (range)	48 (22–94)		48.0 (22–94)		49.0 (26–88)		
Age subgroup, years							
≤ 34	172	6%	162	6%	10	8%	0.100
35–49	1,510	53%	1,455	53%	55	44%	
≥ 50	1,180	41%	1,119	41%	61	48%	
Stage							
I/II	2,457	86%	2,376	87%	81	64%	<0.001
III	405	14%	360	13%	45	36%	
Tumor size, cm							
≤ 2	1,676	59%	1,625	59%	51	40%	<0.001
> 2	1,186	41%	1,111	41%	75	60%	
Lymph node							
Negative	1,749	61%	1,694	62%	55	44%	<0.001
Positive	1,113	39%	1,042	38%	71	56%	
Histologic grade							
1/2	1,787	64%	1,734	65%	53	43%	<0.001
3	1,025	36%	954	35%	71	57%	
Unknown	50		48		2		
Nuclear grade							
1/2	1,775	63%	1,730	64%	45	41%	<0.001
3	1,049	37%	984	36%	65	59%	
Unknown	38		22		16		
LVI							
Negative	2,095	76%	2,007	76%	88	75%	0.830
Positive	660	24%	631	24%	29	25%	
Unknown	107		98		9		
Hormone-receptor status							
Negative	916	32%	831	30%	85	68%	<0.001
Positive^c^	1,940	68%	1,899	70%	41	32%	
Unknown	6		6		0		
Tissue HER2 status							
Negative	2,168	76%	2,146	79%	22	17%	<0.001
Positive^d^	692	24%	588	21%	104	83%	
Unknown	2		2		0		
Definitive surgery							<0.001
Conservation	1,781	62%	1,743	64%	38	30%	
Mastectomy	1,081	38%	993	36%	88	70%	
Radiotherapy							
Yes	2,047	72%	1,973	72%	74	59%	0.001
No	813	28%	761	28%	52	41%	
Unknown	2		2		0		
Chemotherapy							
Yes	1,852	65%	1,746	64%	106	84%	<0.001
No	1,007	35%	987	36%	20	16%	
Unknown	3		3		0		
Anti-HER2 therapy							
Yes	264	9%	211	8%	53	42%	<0.001
No	2,598	91%	2,525	92%	73	58%	

^a^Calculated by chi-square test except where stated otherwise.

^b^Calculated by *t*-test.

^c^Estrogen- and/or progesterone-receptor positive.

^d^Graded as 3+ on IHC or 2+ on fluorescence in situ hybridization in cases of IHC 2+.

**Figure 2:**
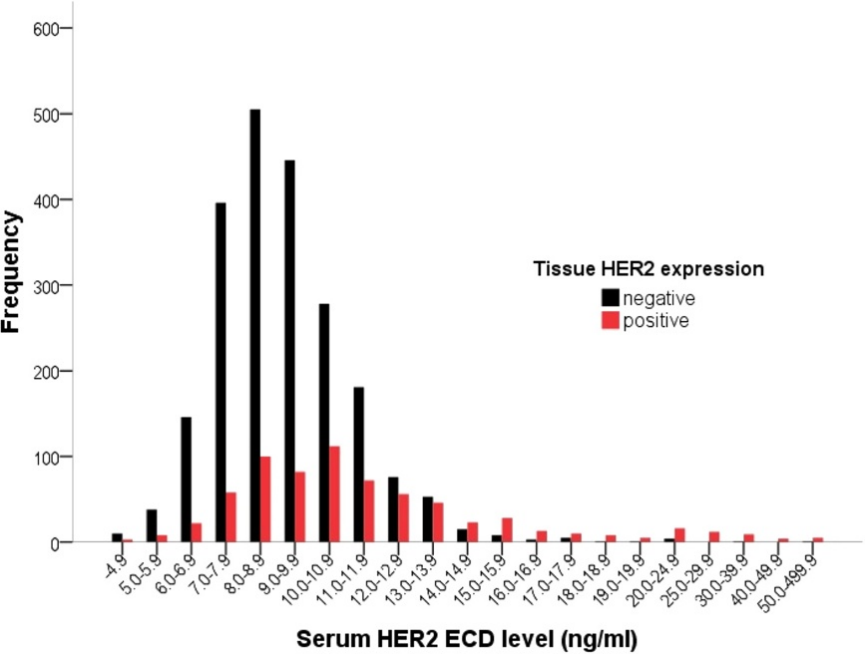
**Distribution of serum HER2 ECD levels according to tissue HER2 status: tissue HER2 positive and negative cases are depicted in red and black, respectively.**

The distribution of preoperative HER2 ECD levels, which ranged from 3.3 to 427.8 ng/ml, shows a positive skew (i.e., the mass of the distribution was concentrated to the left of the histogram), and the presence of outliers at extremely high values (Figure 1). The majority of the extremely high values belonged to the tissue HER2-positive group. When depicted again according to the tissue HER2-expression, it was found that the tissue HER2-positive group had more variable distribution pattern than their tissue HER2-negative counterparts (Table 1 and Figure 2).

Patients with elevated HER2 ECD levels were more likely to have aggressive clinicopathologic variables (Table 2). Age at diagnosis was not related to HER2 ECD levels (Tables 1 and 2). The proportions of patients with elevated ECD level according to various subgroups are summarized in Table 1. In the tissue HER2-positive subgroup, 15.0% of the patients had elevated HER2 ECD, which is more than three times the average (4.4%). Furthermore, the proportion of patients with elevated HER2 ECD levels increased with more advanced and aggressive tumors: 11.3% of those at stage III, 9.3% of those with negative hormone-receptor status, 6.9% of those with high-grade disease, 6.4% of those with positive LN metastasis, and 6.3% of those with a tumor size of >2 cm. Among the 2,168 patients with negative tissue HER2 status, only 22 (1.0%) had a level of HER2 ECD greater than the cutoff value.

### Serum HER2 ECD levels and tissue HER2 status

Of the patients for whom tissue HER2 status could be determined by IHC and/or FISH (*n* = 2,860), 692 (24.2%) had a positive tissue HER2 status. There was a strong statistical correlation between serum HER2 ECD levels and tissue HER2 status in terms of the difference in serum HER2 ECD values (12.8 ± 17.3 and 9.3 ± 4.3 ng/ml, respectively; *P* < 0.001; Table 1) as well as the proportion of patients with elevated serum HER2 ECD level (15.0% and 1.0%, respectively; *P <* 0.001; Table 1) in tissue HER2-positive and -negative subgroups. The overall concordance rate was 78.7%, with a sensitivity of 15.0% and a specificity of 99.0%. In addition, a better sensitivity (54.9%) was obtained when we applied a different cutoff value of 10.2 ng/ml (rather than 15.2 ng/ml), which has been suggested as an alternative cutoff value for Korean populations [31], but the superior specificity (75.3%) and overall concordance rate (70.4%) were not retained [Table 3-(1), (2)].

**Table 3:** **Prediction performance of tissue HER2 status and concordance with serum HER2 ECD level**

Group analyzed ^a^	Cross-table		*P* ^b^	Concordance rate	Prediction performance			
					Sensitivity	Specificity	PPV	NPP
(1) Total patients (*n* = 2,860)			<0.001	78.7	15.0	99.0	82.5	78.5
	Tissue(+)^e^	Tissue(-)^f^						
Elevated^c^	104	22						
Normal^d^	588	2,146						
(2) Total patients (*n* = 2,860) HER2 ECD cutoff = 10.2 ng/ml			<0.001	70.4	54.9	75.3	41.5	84.0
	Tissue(+)	Tissue(-)						
Elevated	380	535						
Normal	312	1,633						
(3) Negative lymph node status (*n* = 1,747)			<0.001	80.3	11.2	99.1	76.4	80.4
	Tissue(+)	Tissue(-)						
Elevated	42	13						
Normal	332	1,360						
(4) Positive lymph node status (*n* = 1,113)			<0.001	76.2	19.5	98.9	87.3	75.4
	Tissue(+)	Tissue(-)						
Elevated	62	9						
Normal	256	786						
(5) Stage I (*n* = 1,243)			<0.001	80.4	9.6	99.2	75.8	80.5
	Tissue(+)	Tissue(-)						
Elevated	25	8						
Normal	236	974						
(6) Stage II (*n* = 1,212)			<0.001	78.7	13.3	98.9	79.2	78.7
	Tissue(+)	Tissue(-)						
Elevated	38	10						
Normal	248	916						
(7) Stage III (*n* = 405)			<0.001	73.3	28.3	98.5	91.1	71.1
	Tissue(+)	Tissue(-)						
Elevated	41	4						
Normal	104	256						

^a^A diagnostic cutoff value of 15.2 ng/ml was used except where stated otherwise.

^b^Calculated by chi-square test.

^c^Elevated serum HER2 ECD level.

^d^Normal serum HER2 ECD level.

^e^Defined as 3+ on IHC or amplification on fluorescence in situ hybridization in cases of IHC 2+.

^f^Tissue HER2 negative.

*PPV* positive predictive value, *NPV* negative predictive value.

**Figure 3:**
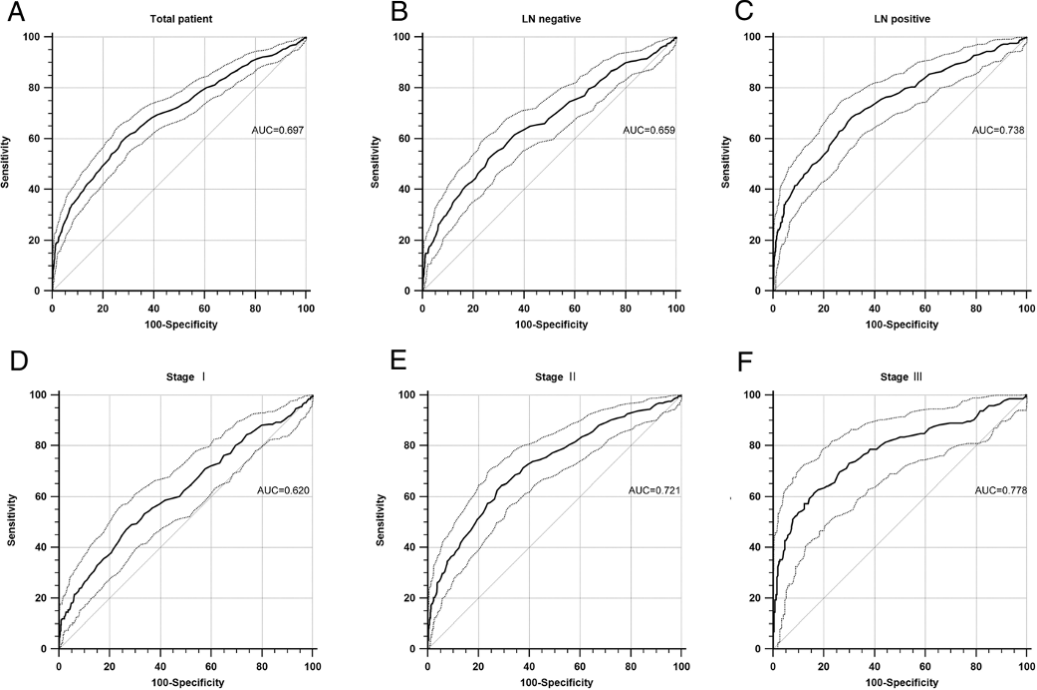
**Receiver operating characteristic (ROC) curves of serum HER2 ECD for predicting the status of tissue HER2 positivity with 95% confidence interval (CI; dotted line). (A)** The area under the ROC curve (AUC) for the entire cohort of 2,860 patients was 0.697 (95% CI = 0.679–0.714, *P* < 0.001). In subgroup analyses, AUCs tended to increase with indexes of the tumor extent: **(B,C)** AUC (95% CI) = 0.659 (0.637–0.682)/0.783 (0.711–0.763) in lymph node (LN)-negative/-positive subgroups; and **(D–F)** AUC (95% CI) = 0.620 (0.592–0.647)/0.720 (0.695–0.746)/0.778 (0.734–0.818) in stage I/II/III subgroups. The *P* values of all ROC curves were <0.001.

As indicated in Figure 3 and Table 3, a series of receiver operating characteristic (ROC) analyses was performed to determine differences in the concordance between the serum and tissue status according to various subgroups, using the cutoff value of 15.2 ng/ml, as used in previous studies [35, 36]. There was a trend toward an improvement in sensitivities and area under the ROC curve (AUC), while retaining good specificities and concordance rates (≥98.5% and ≥73.3% in all subgroups, respectively) with disease status progression (LN negative *vs* positive: sensitivity = 11.2% *vs* 19.5%; stage I *vs* II *vs* III: sensitivity = 9.6% *vs* 13.3% *vs* 28.3%; LN negative *vs* positive: AUC = 0.659 *vs* 0.738; stage I *vs* II *vs* III: AUC = 0.620 *vs* 0.721 *vs* 0.778).

### Prognostic value of serum HER2 ECD level

**Figure 4:**
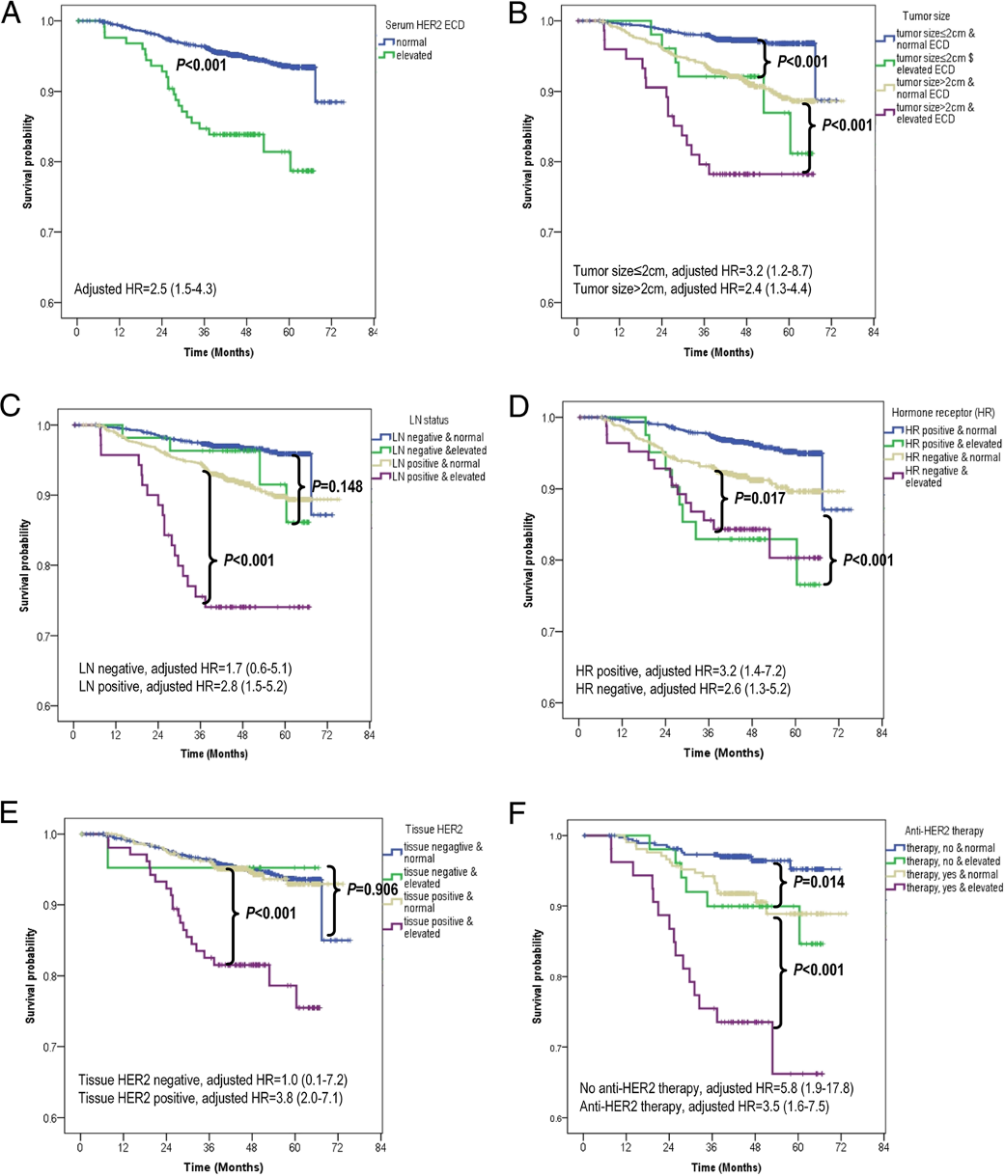
**Distant-metastasis-free survival (DMFS) according to serum HER2 ECD level. (A)** DMFS according to serum HER2 ECD level [elevated level (>15.2 ng/ml) vs normal level (≤15.2 ng/ml)] in the overall series. Elevated serum HER2 ECD level was an independent prognostic factor [log-rank P < 0.001, adjusted hazard ratio (HR) = 2.5, and 95% CI = 1.5–4.3]. **(B–F)** Subgroup analyses by tumor size **(B)**, LN status **(C)**, hormone-receptor status **(D)**, tissue HER2 status **(E)**, and anti-HER2 therapy **(F)**. **(A–F)** Elevated serum HER2 ECD level was significantly prognostic in all subgroups except in those with LN negativity (log-rank *P* = 0.148, adjusted HR = 1.7, 95% CI = 0.6–5.1) and negative tissue HER2 status (log-rank *P* = 0.906, adjusted HR = 1.0, 95% CI = 0.1–7.2). All HRs were adjusted according to the following eight variables: tumor size, LN status, tumor grade, lymphovascular invasion, tissue HER2 status, chemotherapy, antihormone therapy, and trastuzumab therapy.

**Figure 5:**
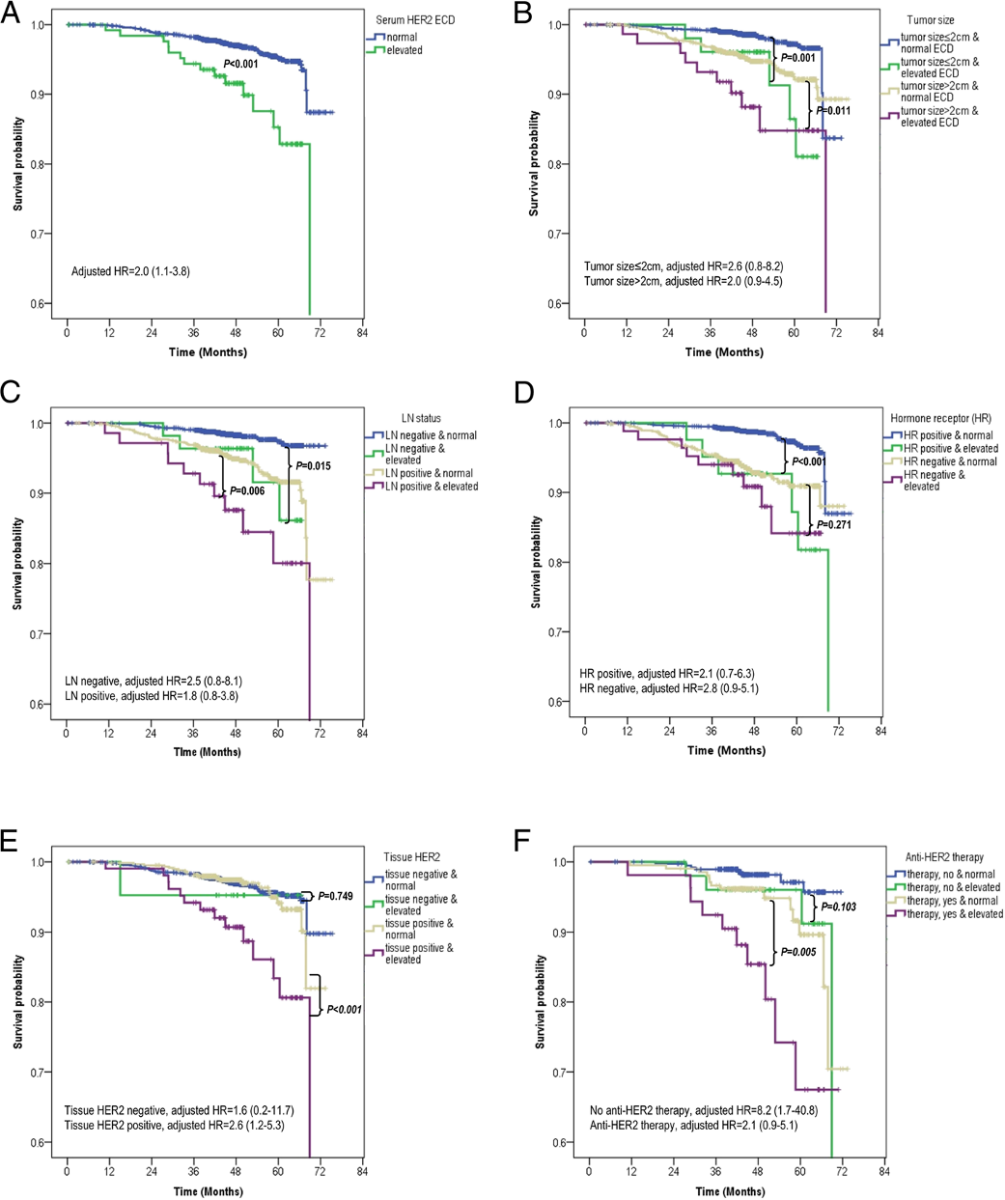
**Breast-cancer-specific survival (BCSS) according to serum HER2 ECD level. (A)** BCSS according to serum HER2 ECD level [elevated (>15.2 ng/ml) vs normal (≤15.2 ng/ml)] in the overall series **(B–F)** Subgroup analyses by tumor size **(B)**, LN status **(C)**, hormone-receptor status **(D)**, tissue HER2 status **(E)**, and anti-HER2 therapy **(F)**. **(A–F)** No adjusted HRs in each subgroup were found to be independently significant, except in the subgroup with positive tissue HER2 status (adjusted HR = 2.6, 95% CI = 1.2–5.3) and nontrastuzumab (adjusted HR = 8.2, 95% CI = 1.7–40.8). All HRs were adjusted according to the following eight variables: tumor size, LN status, tumor grade, lymphovascular invasion, tissue HER2 status, chemotherapy, antihormone therapy, and trastuzumab therapy.

The median follow-up period of all the patients was 46 months (range, 0–75 months). The 5-year distant-metastasis-free survival (DMFS) rate was 81.4% for patients with elevated HER2 ECD and 93.6% for those with normal HER2 ECD (log-rank *P* < 0.001; Figure 4A); and corresponding 5-year breast-cancer-specific survival (BCSS) rates were 85.3% and 95.1% (log-rank P < 0.001; Figure 5A).

Figures 4 and 5 show the results of subgroup analyses. In the log-rank test, an elevated HER2 ECD level (i.e., >15.2 ng/ml) was of prognostic value in terms of DMFS, irrespective of tumor size, hormone-receptor status, and anti-HER2 therapy (Figure 4B,D,F). Furthermore, elevated HER2 ECD level was found to be a significant prognostic factor in subgroups with positive LN status and tissue HER2 overexpression (log-rank *P* < 0.001 and <0.001, respectively), but not in those with negative LN and negative tissue HER2 status (log-rank *P* = 0.148 and 0.906, respectively; Figure 4C,-E). Although in terms of BCSS, elevated HER2 ECD levels were significant in all 2,862 patients (log-rank *P* < 0.001) and in subgroups with any tumor size, with any LN status, with positive hormone-receptor status, and with positive tissue HER2 status, adjusted hazard ratios (HRs) calculated for each subgroup revealed none to be independently significant, except in a subgroup with positive tissue HER2 status [log-rank *P* < 0.001, adjusted HR = 2.6, 95% confidence interval (CI) = 1.2–5.3; Figure 5E]. Adjusted HRs of an elevated HER2 ECD level (depicted in Figures 4 and 5) showed the same trend of prognostic significance as in the log-rank test. Statistically adjusted prognostic values in subgroups according to anti-HER2 therapy in patients with positive tissue HER2 expression were observed significant in both subgroups in terms of DMFS, but only in the nontrastuzumab subgroup in terms of BCSS (Figures 4F and 5F).

**Figure 6:**
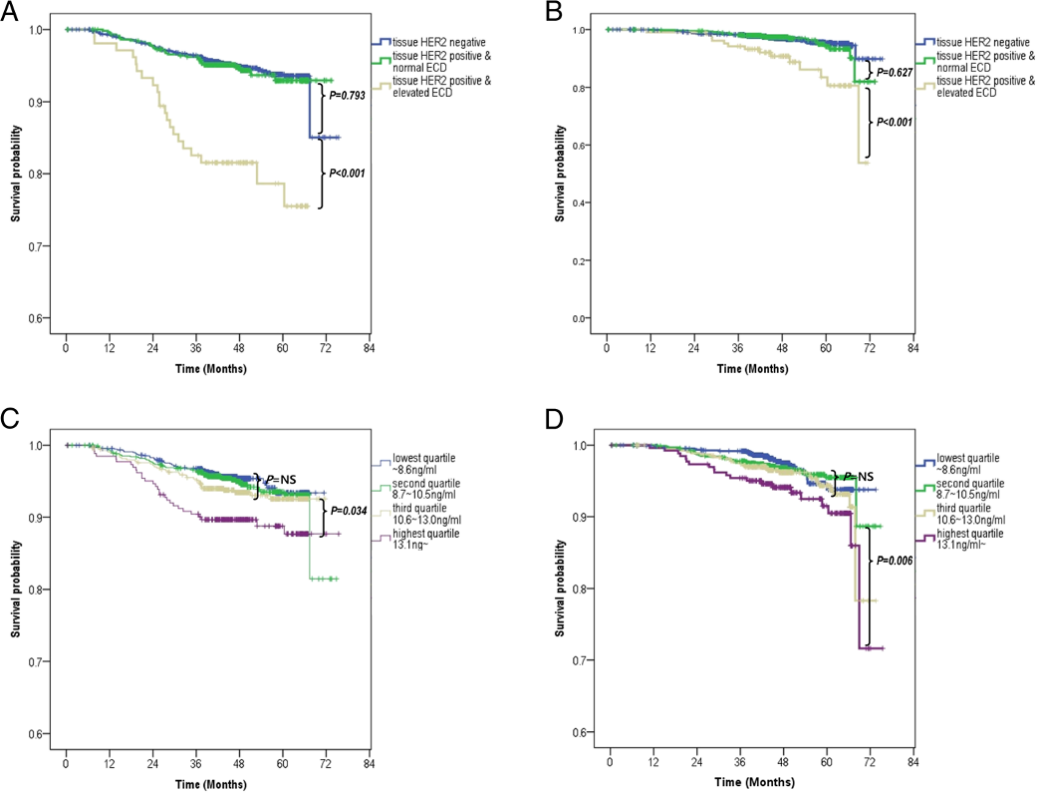
**Distant-metastasis-free survival (DMFS) and** **breast-cancer-specific survival (BCSS) according to HER2 status in both tissue and serum.** DMFS **(A)** and BCSS **(B)** among three subgroups classified according to both tissue and serum HER2 status: tissue– irrespective of serum status, tissue+/serum–, and tissue+/serum+. **(C,D)** There was a significant trend toward a reduction in survival with increasing serum HER2 ECD values in only 692 patients with a positive tissue HER2 status.

We classified all 2,860 patients into 3 subgroups according to HER2 status [i.e., positive (+) and negative (-)] in both tissue and serum: tissue–, irrespective of serum status (*n* = 2,168), tissue+/serum– (*n* = 588), and tissue+/serum + (*n* = 104). As shown in Figure 6, despite tissue HER2 overexpression being a significant prognostic factor in all patients for DMFS and BCSS (log-rank *P* = 0.015 and 0.035, respectively; data not shown), comparison of the three subgroups revealed that the DMFS and BCSS did not differ between the tissue+/serum– and tissue– subgroups (*P* = 0.793 and 0.627, respectively; Figure 6A,B). The tissue+/serum + subgroup had the most ominous prognosis, which suggests a significant role of serum HER2 ECD level as a prognosticator in the tissue HER2-positive patients. In addition to applying the dichotomizing cutoff value of 15.2 ng/ml, four subgroups categorized by serum HER2 ECD levels, only in 692 patients with positive tissue HER2 status, exhibited a trend toward a dose–response relationship in terms of prognosis: higher serum HER2 ECD levels were associated with worse DMFS and BCSS (Figure 6C,D).

The two Cox proportional-hazards models given in Table 4 suggested that HER2 ECD level was a robust independent prognostic factor in patients with tissue HER2-positive breast cancer, irrespective of the variable tentative cutoff values: elevated *v*s normal (HR = 3.788, 95% CI = 2.034–7.058, *P* < 0.001, Table 4) and third/highest quartile *vs* lowest quartile (third quartile: HR = 3.029, 95% CI = 1.003–9.152, *P* = 0.049; highest quartile: HR = 4.746, 95% CI = 1.591–14.160, *P* = 0.005; Table 4).

**Table 4:** **Cox proportional-hazards regression model for distant-metastasis-free survival (DMFS) and breast-cancer-specific survival (BCSS) in 692 patients with tissue HER2-positive breast cancer**

Variable	DMFS			BCSS		
	HR	95% CI	*P*	HR	95% CI	*P*
**Model with dichotomized serum HER2 ECD level, elevated vs normal**						
Serum HER2 ECD level, >15.2 ng/ml	3.788	2.034–7.054	<0.001^a^	2.564	1.230–5.343	0.012^a^
Tumor size, >2 cm	1.731	0.859–3.487	0.125	1.134	0.515–2.497	0.754
Lymph node positive	2.614	1.011–6.756	0.047^a^	2.428	0.812–7.257	0.112
Grade 3+	1.972	1.069–3.638	0.030^a^	1.575	0.758–3.270	0.223
Lymphovascular invasion	2.625	1.419–4.855	0.002^a^	2.740	1.315–5.709	0.007^a^
Chemotherapy, yes	0.235	0.081–0.687	0.008^a^	0.303	0.085–1.078	0.065
Hormone therapy, yes	1.576	0.841–2.951	0.156	1.032	0.494–2.156	0.933
Trastuzumab, received	1.734	0.902–3.332	0.099	1.969	0.903–4.292	0.089
**Model with four subgroups classified by serum HER2 ECD levels, each quartile group vs lowest-quartile subgroup**						
Serum HER2 ECD level,						
Lowest quartile (reference)	1			1		
Second quartile	1.827	0.549–6.078	0.326	1.008	0.224–4.529	0.992
Third quartile	3.029	1.003–9.152	0.049^a^	2.407	0.669–8.655	0.179
Highest quartile	4.746	1.591–14.160	0.005^a^	2.826	0.785–10.176	0.112
Tumor size, >2 cm	1.741	0.864–3.507	0.121	1.146	0.519–2.534	0.736
Lymph node positive	2.502	0.982–6.376	0.055	2.407	0.804–7.211	0.117
Grade 3+	1.844	1.010–3.370	0.046^a^	1.844	1.010–3.370	0.046^a^
Lymphovascular invasion	2.303	1.250–4.240	0.007^a^	2.550	1.223–5.317	0.012^a^
Chemotherapy, yes	0.210	0.074–0.599	0.004^a^	0.273	0.078–0.957	0.042^a^
Hormone therapy, yes	1.443	0.784–2.655	0.238	1.038	0.501–2.150	0.920
Trastuzumab, received	1.930	1.013–3.677	0.045^a^	2.098	0.968–4.549	0.061

^a^Significant at *P* < 0.05.

## Discussion

The present study found that serum HER2 ECD level was correlated with a poor prognosis in primary breast cancer, and that patients with an elevated HER2 ECD level (>15.2 ng/ml) had a worse DMFS and BCSS than those with normal HER2 ECD levels. In particular, it was confirmed that its role as an independent prognostic factor was clinically robust at various tentative cutoff values in patients with positive tissue HER2 status.

**Figure 7:**
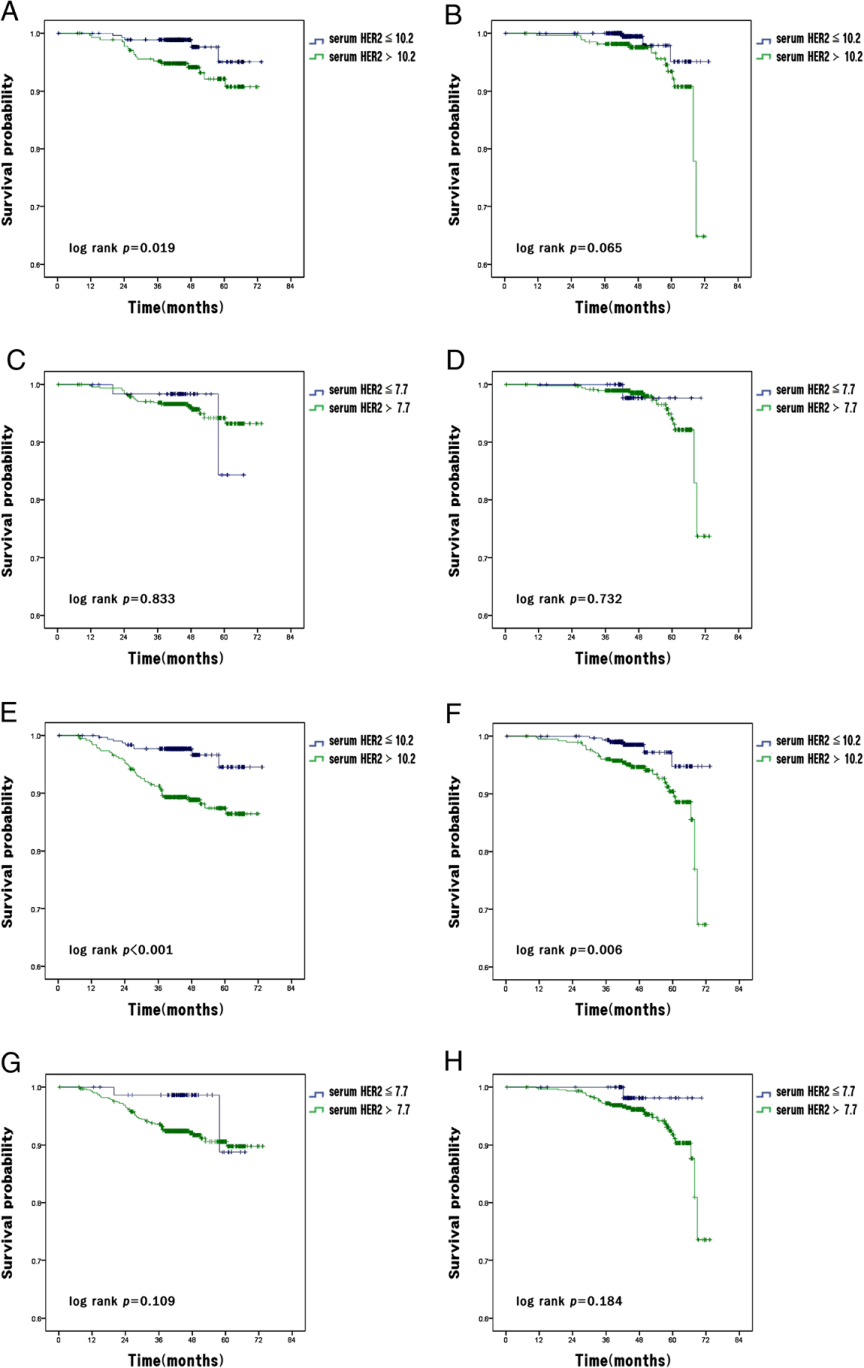
**Distant-metastasis-free survival (DMFS) and Breast-cancer-specific survival (BCSS) according to the serum HER2 ECD level by the lower cutoffs and the early stages. (A)** DMFS according to serum HER2 ECD level [elevated level (>10.2 ng/ml) vs normal level (≤10.2 ng/ml)] in breast cancer patients with stage I and II. **(B)** BCSS according to serum HER2 ECD level [elevated level (>10.2 ng/ml) vs normal level (≤10.2 ng/ml)] in breast cancer patients with stage I and II **(C)** DMFS according to serum HER2 ECD level [elevated level (>7.7 ng/ml) vs normal level (≤7.7 ng/ml)] in breast cancer patients with stage I and II **(D)** BCSS according to serum HER2 ECD level [elevated level (>7.7 ng/ml) vs normal level (≤7.7 ng/ml)] in breast cancer patients with stage I and II **(E)** DMFS according to serum HER2 ECD level [elevated level (>10.2 ng/ml) vs normal level (≤10.2 ng/ml)] in breast cancer patients with stage I, II and III **(F)** BCSS according to serum HER2 ECD level [elevated level (>10.2 ng/ml) vs normal level (≤10.2 ng/ml)] in breast cancer patients with stage I, II and III **(G)** DMFS according to serum HER2 ECD level [elevated level (>7.7 ng/ml) vs normal level (≤7.7 ng/ml)] in breast cancer patients with stage I, II and III **(H)** BCSS according to serum HER2 ECD level [elevated level (>7.7 ng/ml) vs normal level (≤7.7 ng/ml)] in breast cancer patients with stage I, II and III.

There is still no general consensus as to a definitive dichotomizing HER2 ECD cutoff value for clinical use, so we adopted the most commonly used value of 15.2 ng/ml to define elevated HER2 ECD. HER2 ECD levels were previously reported to be elevated in 11.4% (range, 3.1–34.1%) of primary breast cancer patients and 36.5% (range, 23–62%) of metastatic breast cancer patients [34]. In the present study, elevated HER2 ECD levels were observed in 4.4% (126/2,862) of the primary breast cancer patients, which is a noteworthy finding because it was derived from a large number of patients at a single institute and using a single US FDA-approved assay. In addition, as shown in Table 1, significant differences in the prevalence of elevated HER2 ECD levels between the subgroups were confirmed, which is in accordance with previous results [30, 36]. And, other studies have suggested that setting a lower cutoff value such as 7.7 ng/ml and 10.2 ng/ml or analyzing early stage patients could come with a better outcome, however our additional study adjusted with these did not show a better result (Figure 7). However, it should be noticed that, our analysis did not address the issues on more optimal cutoff values for HER2 ECD raised by previous studies [31, 38, 39].

Interestingly, elevated serum HER2 ECD levels were detected in 22 (1.0%) of the 2,168 tissue HER2-negative patients (26.4 ± 35.6 ng/ml; range, 15.3–184.4 ng/ml). Possible explanations for this are chance false-positivity, normal elevation of serum HER2 ECD level as reported in healthy controls [40], a minority of HER2-positive cells being lower than the definition of tissue HER2 positivity as its source [41], and genetic differences between individuals with respect to matrix metalloproteinase activity, which is responsible for HER2 release into the serum [39]. Since metastasis had occurred in only 1 of the 22 patients in this subgroup, the clinical implication of unexpected serum ECD elevation could not be further investigated.

In this study we observed a significant association between elevated HER2 ECD level and the parameters of tumor aggressiveness (Table 2). More patients had undergone mastectomy among those with an elevated HER2 ECD levels than in those with a normal HER2 ECD level. This might be caused by the tumor characteristics being more aggressive in the former subgroup than in the latter, the cause of which was not fully established.

The association between serum HER2 ECD concentration and tissue HER2 status remains controversial in primary breast cancer, which may be attributable to the use of different cutoff values, small samples, and different patient populations in the various studies. For now it seems reasonable to suggest that serum HER2 ECD levels cannot substitute for tissue HER2 expression, but can provide additional information. The aim of the present study was to compare the concordances according to the subgroups and provide practical information when we suspected the inherent imperfection of tissue HER2 results in the case of elevated serum HER2 ECD levels. We applied two cutoff values: 15.2 ng/ml, which has been approved by the US FDA; and 10.2 ng/ml, which has been suggested as a more appropriate cutoff value for the Korean population [31]. Although a lower cutoff value could yield a higher sensitivity and negative predictive value (Table 3), it negatively affected the specificity and positive predictive value. In our opinion, in view of the adjunctive role of serum HER2 ECD level, the cutoff of 15.2 ng/ml may have greater clinical utility because higher specificity and positive predictive values are mandatory to reduce the likelihood of false suspicion of tissue HER2 status due to the abnormal serum HER2 ECD level. Unlike previous studies [35, 36], the present findings suggest that significant correlations between the expression of tissue HER2 and serum HER2 ECD level exist in primary breast tumor, regardless of whether the primary tumor is early or advanced (Table 3). However, the finding that AUCs increased as the indexes of tumor extent increased should not be overlooked (Figure 3).

Despite the relatively short follow-up, the inclusion of a large sample enabled us to demonstrate the poor prognostic role of HER2 ECD level. Moreover, we determined that tissue HER2 status had no prognostic impact on either DMFS or BCSS in patients with a normal serum HER2 ECD level. Thus, the serum HER2 ECD level can be a valuable prognostic factor in patients with primary breast cancer with tissue HER2 overexpression (Figure 6 and Table 4). The persistent adverse prognostic value of elevated HER2 ECD levels in stage I/II early breast cancer could reflect the presence of micrometastases or high rates of HER2 cleavage and shedding, with the production of truncated cell-associated fragments that contain the signaling kinase domain that is activated in the absence of the ECD. As a result, these tumors with a deregulated growth-promoting pathway could behave more aggressively [30].

To clarify the possibility of false-positive results in the present study in terms of prognosis according to the arbitrary cutoff value of 15.2 ng/ml, we validated its prognostic role among continuous subgroups classified relative to the quartile values of serum HER2 ECD levels in tissue HER2-positive tumors: the lowest-quartile, median, and highest-quartile values were 8.7, 10.6, and 13.1 ng/ml, respectively. The Kaplan-Meier curves (Figure 6C, D) and multivariate analysis revealed that the third- and highest-quartile subgroups independently had an ominous prognosis in terms of DMFS (third quartile: HR = 3.029, 95% CI = 1.003–9.152, *P* = 0.049; highest quartile: HR = 4.746, 95% CI = 1.591–14.160, *P* = 0.005; Table 4).

The possibility of an association between HER2 ECD level and trastuzumab response was assessed in 264 patients receiving trastuzumab and in 428 patients without trastuzumab administration. We found that HER2 ECD level was a poor prognostic indicator, irrespective of the treatment in terms of DMFS (Figure 4F). In terms of BCSS, there did not appear to be a significant prognostic role of elevated HER2 ECD level in the trastuzumab subgroup (Figure 5F). This lack of a significant finding may be attributable to the relatively short follow-up. Also, only 38.2% (264/692) of patients with HER2-positive tumors had been treated with trastuzumab in the cohort. It is partly related to the inclusion of patients with small tumors. Our study did not include patients receiving neoadjuvant systemic therapy. More importantly, the use of trastuzumab in the adjuvant setting was just covered for advanced breast cancer by the Korean national health insurance during the period of this study (2007 ~ 2009). In patients with tissue HER2 positive, T1 was 54.5% (377/692), and N0 was 54.0% (374/692), which needs to be taken into consideration. Due to the nature of a retrospective analysis without randomization, it was not possible to compare the responses or benefits of trastuzumab among the subgroups of 261 patients who received this drug with elevated (*n* = 53) and normal (*n* = 208) HER2 ECD levels.

While the present study is subject to the same limitations as other retrospective studies, it provides robust evidence supporting the valuable clinical utility of HER2 ECD level, drawn successfully because of the inclusion of a large sample. The presented results indicate that a preoperatively elevated HER2 ECD level reflects tumor extension, with significantly higher values being found in patients with larger tumors, with LN metastasis, or with LVI, and that patients with an elevated HER2 ECD level are more likely to develop distant metastasis irrespective of their disease status.

## Conclusions

Given the prevalence of HER2 ECD elevation, preoperative serum HER2 ECD measurement can be clinically useful in patients with tissue HER2-positive primary breast cancer.

## Abbreviations

BCSS: Breast-cancer-specific survival
DMFS: Distant-metastasis-free survival
ECD: Extracellular domain
FISH: Fluorescence *in situ* hybridization
HER2: Human epidermal growth factor receptor-2
IHC: Immunohistochemistry.
